# Sensitization of glioblastoma cells to TRAIL-induced apoptosis by IAP- and Bcl-2 antagonism

**DOI:** 10.1038/s41419-018-1160-2

**Published:** 2018-11-01

**Authors:** Frank A. Lincoln, Dirke Imig, Chiara Boccellato, Viktorija Juric, Janis Noonan, Roland E. Kontermann, Frank Allgöwer, Brona M. Murphy, Markus Rehm

**Affiliations:** 1 Department of Physiology and Medical Physics, Dublin 2, Ireland; 2 Centre for Systems Medicine, Royal College of Surgeons in Ireland, Dublin 2, Ireland; 3 Institute for Systems Theory and Automatic Control, University of Stuttgart, Stuttgart, Germany; 4 Institute of Cell Biology and Immunology, University of Stuttgart, Stuttgart, Germany; 5 Stuttgart Research Center Systems Biology, University of Stuttgart, Stuttgart, Germany

## Abstract

Due to the lack of effective treatments for glioblastoma (GBM), we here studied the responsiveness of GBM cell lines to the combination of death ligand, TRAIL and the IAP antagonist, TL32711 (Birinapant). Responses were highly heterogeneous, with synergistic apoptosis as well as treatment resistance observed. Caspase-8 and Bid, together with caspase-3, form a nonlinear signalling hub that efficiently induced apoptosis in responder cell lines. Cells resistant to TRAIL/TL32711 expressed low amounts of procaspase-8 and Bid and poorly activated caspase-3. We therefore hypothesised that improving caspase-8 activation or sensitising mitochondria to truncated Bid (tBid) could convert non-responder GBM cell lines to responders. Mathematical simulations of both strategies predicted mitochondrial sensitization to tBid would outperform enhancing caspase-8 activation. Indeed, antagonising Bcl-2 by ABT-199 allowed TRAIL/TL32711 response synergies to manifest in otherwise TRAIL resistant cell lines. These findings were further corroborated in experiments with a translationally relevant hexavalent TRAIL variant. Our study therefore demonstrates that a high caspase-8/Bid signature is associated with synergistic TRAIL/TL32711-induced apoptosis in GBM cells and outlines Bcl-2 antagonism as a highly potent intervention to sensitize highly TRAIL-resistant GBM cells to TRAIL/TL32711 combination treatment.

## Introduction

Glioblastoma (GBM) is the most common aggressive brain tumour, with currently no effective therapies being available. Standard-of-care includes surgery, followed by DNA-damaging radiotherapy and chemotherapy, in the hope to eliminate tumour cells by triggering apoptotic cell death. However, median survival remains at only 14.6 months and many patients do not benefit from these therapies at all^1,2^. Reasons for the poor responsiveness to these therapies include effective DNA repair as well as inactivating mutations in tumour suppressor p53, the primary transcription factor to induce apoptosis in response to DNA damage^3,4^. It is therefore highly relevant to explore if transcription-independent cell death programs, such as extrinsic death-receptor mediated apoptosis, can be induced efficiently in GBM. Of particular interest is tumour necrosis factor-related apoptosis-inducing ligand (TRAIL), which preferably induces caspase-8 dependent apoptosis in cancer cells, but not in untransformed cells, by activating its cognate death receptors TRAIL-R1 (DR4) and TRAIL-R2 (DR5)^5,6^. However, cell death responses to TRAIL and first generation TRAIL-based therapeutics remain heterogeneous or poor in most cancers^5,7^, with GBM cell lines being particularly resistant to TRAIL^8,9^. Besides strategies to optimize receptor ligands^10–12^, antagonizing pro-survival proteins could be a viable strategy to enhance cell death signaling in response to TRAIL. Of particular interest are antagonists of inhibitor of apoptosis proteins (IAPs), such as TL32711 (Birinapant), a bivalent IAP antagonist in phase 2 clinical trials for single and combination treatments of various cancers (clinicaltrials.gov). TL32711 binds to cellular IAPs (cIAPs) 1 and 2 with high affinity and causes their rapid degradation at nM concentrations^13^. cIAPs suppress apoptosis by recruiting the components of the linear ubiquitin chain assembly complex (LUBAC) to complex 1, also known as the death-inducing signaling complex, a signaling platform comprising of oligomerised death receptors and the adapter protein Fas-associated death domain (FADD). LUBAC activity promotes NFκB activation and pro-survival signaling^14^. cIAPs also suppress caspase-8 activation and initiation of receptor-interacting serine/threonine-protein kinase 1 (RIPK1)-dependent apoptosis and necroptosis on subsequently forming cytosolic FADD-containing signaling platforms (complex 2)^11,14,15^. On both complexes 1 and 2, caspase-8 activation is further regulated by splice variants of the inactive caspase-8 homolog cFLIP, with high amounts of cFLIP_L_ and cFLIP_S_ inhibiting apoptosis^16,17^. Caspase-8 proteolytically activates two key pro-apoptotic substrates, the BH3-only protein Bid as well as effector caspase-3, with the latter likewise being able to activate Bid. Apoptosis through direct caspase-3 activation requires very high amounts of caspase-8 and/or absence of caspase-3 inhibitors^18,19^. Truncated Bid (tBid), instead, activates Bax and Bak, thereby triggering signaling cascades that strongly amplify caspase-3 activation to ensure efficient apoptosis execution^20^. The threshold for whether tBid can induce Bax/Bak-dependent apoptosis execution is set by the amounts of anti-apoptotic Bcl-2 family members, such as Bcl-2 itself^20^.

Here, we describe that GBM cell lines respond heterogeneously to the combination of TRAIL and IAP antagonist TL32711. Responsiveness depends on efficient and non-linear signal transduction through a pre-mitochondrial pro-apoptotic signaling hub that comprises caspase-8, caspase-3 and Bid. Based on a systems biology approach, we identified an efficient strategy to sensitize non-responding cell lines to TRAIL/TL32711 and provide proof-of-concept that our findings are transferable to studies, in which translationally relevant 2nd generation hexavalent TRAIL receptor agonists are used.

## Results

### GBM cells respond heterogeneously to the combination of TRAIL and IAP antagonist TL32711

**Fig. 1:**
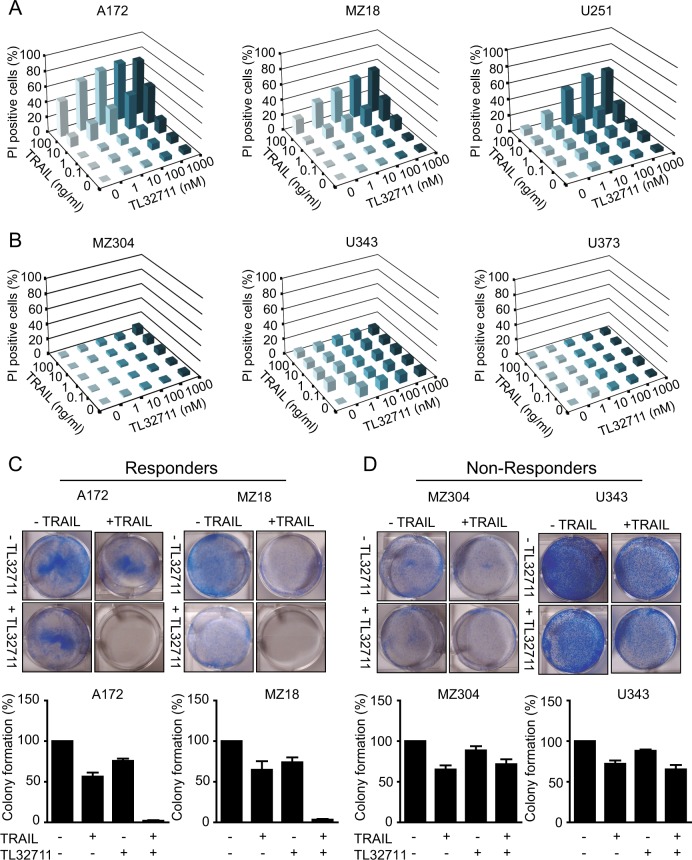
GBM cell lines respond heterogeneously to the combination of TRAIL/ TL32711. **a**, **b** GBM cell lines were treated with TRAIL, TL32711 alone and in combination for 24 h at the indicated concentrations. Cell death was measured by PI-based semi-HTS flow cytometry and shown as increase above untreated controls. Data are shown as means of three independent experiments, with triplicate samples analysed in each experiment. Error bars (SEM) were typically <10% and were omitted for clarity. **a** GBM cell lines that responded synergistically towards the combined treatment of TRAIL and TL32711 (Responders). **b** GBM cell lines that remained largely resistant to the combined treatment of TRAIL and TL32711 (non-responders). **c**, **d** Colony formation capacity was tested in responder and non-responder cell lines following 24 h of treatment (responders: 10 ng/ml TRAIL, 100 nM TL32711; non-responders: 100 ng/ml TRAIL, 1 µM TL32711). After 10 days of growth, colonies were stained with crystal violet and counted. Representative micrographs are shown. Bar graphs show mean ± SEM from three independent experiments

To obtain an overview of the responsiveness of GBM cells to human recombinant TRAIL, to IAP antagonist TL32711 or the combination thereof, we studied a panel of six commercially available or early passage GBM cell lines. Cell death was measured at 25 treatment conditions to obtain a comprehensive overview of response heterogeneities, using propidium iodide uptake as a marker of plasma membrane permeabilization. With the exception of A172 cells, all cell lines were highly resistant to TRAIL as a single agent (Fig. 1a, b). Similarly, all cell lines were resistant to TL32711 (Fig. 1a, b). Interestingly, though, the subset of A172, MZ18 and U251 cells responded highly synergistically to the combination of TRAIL and TL32711 in a dose dependent manner (40–80% cell death) (Fig. 1a). Response synergies were confirmed by Webb’s fractional product calculations, with highest synergies (scores of 0.55, 0.57 and 0.51) determined for A172, MZ18 and U251, respectively. In contrast, MZ304, U343 and U373 cells remained fully resistant (Fig. 1b). These groups of cell lines are hereafter designated as responders and non-responders, respectively. To complement the cell death screen, we assessed the effect of single and combination treatments on long-term survival and clonogenicity of selected responder and non-responder cell lines. Only in responders, the combination of TRAIL and TL32711 drastically reduced clonogenic survival (Fig. 1c, d). Together, these findings demonstrate that the combination of TRAIL and TL32711 results in synergistic cell lethality, but only in 50% of the tested cell lines.

### Apoptosis is the primary cell death modality in response to TRAIL/TL32711 in GBM cells

**Fig. 2:**
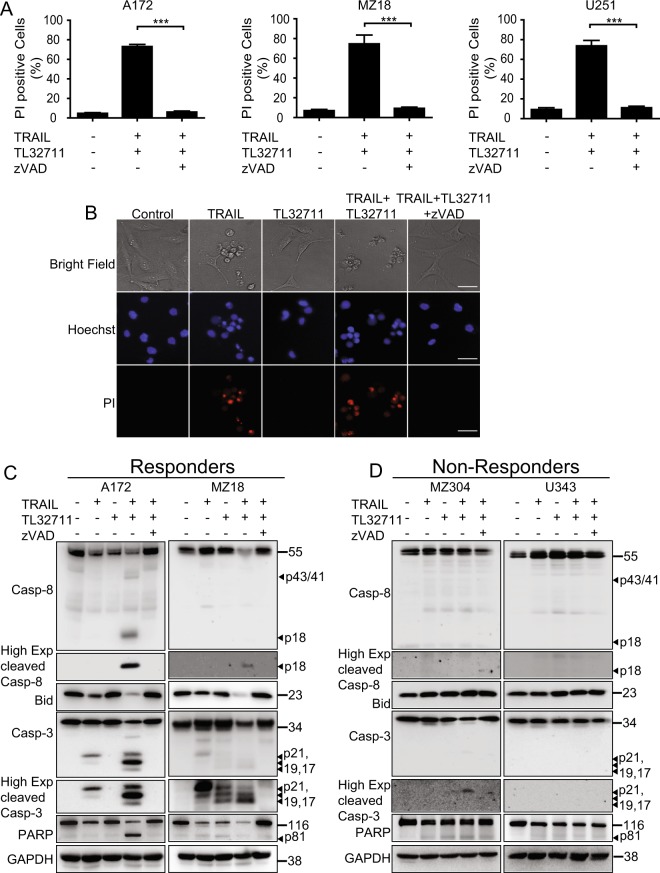
Apoptosis is the primary cell death modality induced by the combination of TRAIL/TL32711. **a** Responder cell lines were treated with the combination of TRAIL/TL32711 in the presence or absence of pan-caspase inhibitor zVAD-fmk (50 µM) for 24 h. Cell death was measured by PI-based flow cytometry. Data represent mean ± SEM of three independent experiments. ***p < 0.001 (one-way ANOVA followed by Tukey post hoc test). **b** Representative micrographs of A172 cells after 24 h of treatment. Addition of zVAD-fmk prevented cell death (PI staining), nuclear condensation and fragmentation (Hoechst 33342 staining) and morphological changes indicative of apoptotic cell death. **c**, **d** Caspase processing and substrate cleavage can be detected in responders but not in non-responders after 24 h of treatment (responders: 100 ng/ml TRAIL, 100 nM TL32711; non-responders: 100 ng/ml TRAIL, 1 µM TL32711). Whole-cell lysates were analysed for the indicated proteins by western blotting. GAPDH served as loading control. Representative western blots from three independent experiments are shown

As TRAIL receptor activation might not only induce apoptosis but possibly also necroptosis^21^, we next studied which type of cell death dominates in GBM cells responsive to TRAIL/TL32711. In all responder cell lines, cell death occurred entirely by apoptosis, since broad-range caspase inhibitor zVAD-fmk prevented PI-positivity (Fig. 2a). Correspondingly, pharmacological inhibition of RIP1 with necrostatin-1 did not affect cell death responsiveness, with the exception of modest reductions in cell death in MZ18 cells, indicating a contribution of RIP1-dependent apoptosis in this cell line^15^ (Supplementary Fig. 1). Nuclear morphologies of dying cells confirmed these conclusions, with prominent nuclear condensation and fragmentation observed (Fig. 2b). Apoptosis induction was furthermore confirmed by processing of procaspases-8 and -3, and by cleavage of key caspase substrates. In responders, TRAIL/TL32711 combination treatment acted in concert to trigger enhanced processing of the caspase zymogens and accumulation of active subunits, such as caspase-8 p18 and caspase-3 p19/17 (Fig. 2b). Similarly, loss-of-full length Bid and PARP was most pronounced for TRAIL/TL32711 combination treatments (Fig. 2b). In contrast, active caspase subunits or cleaved substrates were not detected in notable amounts in non-responders (Fig. 2c). IAP antagonists were reported to induce TNFα secretion in some cell lines, a process that can induce apoptosis through TNF-receptor activation^22,23^. However, we could not detect secreted TNFα in supernatants collected from responder or non-responder cell lines (A172, MZ18 and MZ304, U343, respectively) at baseline or after treatment with TRAIL, TL32711 or the combination thereof (not shown). TRAIL/TL32711-induced apoptosis therefore proceeds without a requirement for autocrine TNFα signalling towards cell death.

### Post-treatment IAP regulation and residual, sub-lethal caspase activation in non-responders

**Fig. 3:**
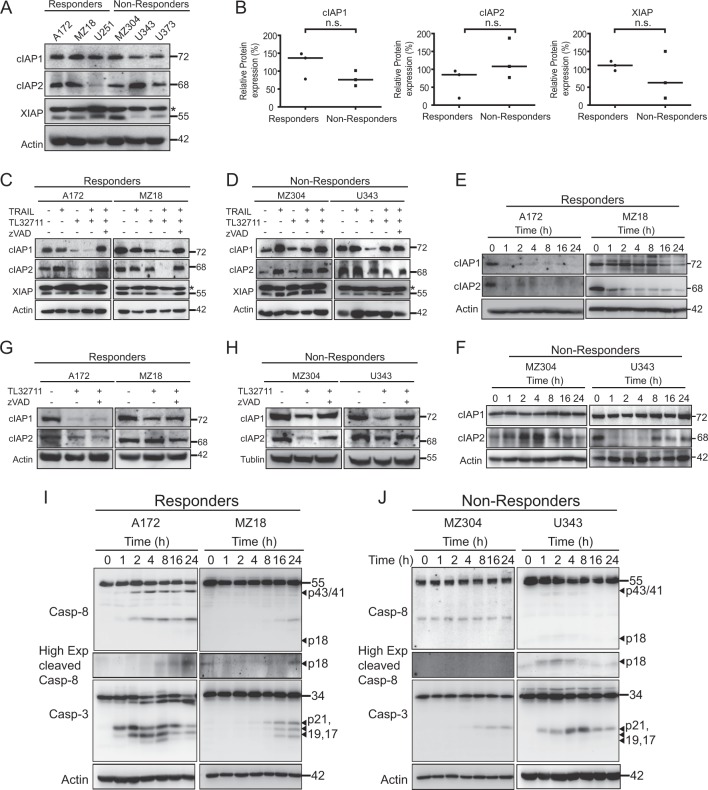
Post-treatment IAP regulation and residual, sub-lethal caspase activation in non-responders. **a** Basal expression level of IAP proteins analysed from whole-cell lysates by western blotting. Actin served as loading control. **b** Protein amounts were quantified from original 12 bit grey scale images and compared between responder and non-responder cell lines. Lines represent medians. n.s., not significant (Mann–Whitney *U*-test). **c**, **d** IAP amounts following 24 h of treatment (responders: 100 ng/ml TRAIL, 100 nM TL32711; non-responders: 100 ng/ml TRAIL, 1 µM TL32711; 50 µM zVAD-fmk). **e**, **f** Temporal profiles of cIAP1 and cIAP2 in responder and non-responder cell lines. Actin served as loading control. **g**, **h** Responder and non-responder cell lines were exposed to 1 µM TL32711 in the presence or absence of zVAD-fmk (50 μM) for 24 h. cIAP1 and cIAP2 protein amounts were assessed by western blotting. Actin or tubulin served as loading control. **i** Caspase processing kinetics in responder cell lines. Cells were treated with TRAIL (100 ng/ml) and TL32711 (100 nM). **j** Residual or transient caspase processing in non-responder cell lines. Cells were treated with TRAIL (100 ng/ml) and TL32711 (1 µM). Actin served as loading control. Representative western blots from three independent experiments are shown

Next, we studied whether basal IAP amounts allow separating responder from non-responder GBM cell lines. Quantitative immunoblotting showed considerable variance in cIAP2 and XIAP expression across the cell lines, whereas cIAP1 was expressed similarly (Fig. 3a). Expression amounts, however, did not differ significantly between responders and non-responders (Fig. 3b). A loss of both cIAP1 and cIAP2 was described for cell lines highly responsive to IAP antagonists^22,23^, but in many cases, including glioma, changes in IAP amounts and treatment responsiveness can be highly heterogeneous^24^. Furthermore, activation of non-canonical NFκB signalling in response to IAP antagonists can lead to the recovery or accumulation of cIAPs, in particular cIAP2^25^. TRAIL likewise can induce NFκB signalling, resulting in altered protein expression patterns^11^. We therefore studied IAP amounts after treatment with TRAIL, TL32711 and TRAIL/TL32711. In additional controls, zVAD-fmk was added to account for potential caspase-dependent IAP degradation. We observed heterogeneous changes in IAP amounts. In A172 cells, TRAIL-induced increases in cIAP2, whereas TL32711 and the combination of TRAIL/TL32711 drastically reduced the amounts of cIAP1 and cIAP2. XIAP loss was observed for the combination treatment and could be rescued by zVAD-fmk (Fig. 3c), in line with XIAP being an effector caspase substrate^26^. Caspase inhibition also restored the amounts of cIAP1 and cIAP2 in responder cell line A172 (Fig. 3c). Similar findings on IAP recovery were made in MZ18 cells. In these cells, though, TL32711 was far less potent at reducing cIAP2 amounts (Fig. 3c). In the non-responder cell line MZ304, cIAPs accumulated upon TRAIL addition (Fig. 3d). In U343 cells, TL32711 potently reduced cIAP1 and cIAP2 amounts, whereas in combination treatments only cIAP2 was downregulated (Fig. 3d). XIAP amounts remained unchanged, in line with a lack of effector caspase activity in non-responders. A kinetic analysis of cIAP changes upon TRAIL/TL32711 treatment highlighted additional differences between responders and non-responders. Responder cell lines rapidly lost cIAP2 and presented reductions in cIAP1 (Fig. 3e). In non-responder cell lines, an initial small decline in cIAP1 was followed by recovery to pre-treatment amounts (Fig. 3f). Differences meanwhile were observed in cIAP2 levels in the non-responder cell lines. In MZ304 cells, cIAP2 increased transiently to then drop below basal expression amounts at the endpoint (Fig. 3f). In U343 cells, cIAP2 decreased transiently, but failed to fully recover (Fig. 3f).

Interestingly, we found that TL32711-induced decreases in cIAPs in non-responder cell lines might depend on caspase activation, as zVAD-fmk addition restored cIAP amounts in these cell lines (Fig. 3g, h). Whereas, we did not detect notable caspase activation in non-responders by endpoint analysis (Fig. 2d), these cells might activate caspases only transiently and sub-lethally. We therefore performed kinetic studies of caspase processing. Responder cell lines processed procaspases-8 and -3 with notably different kinetics, but with significant amounts of fully processed caspase subunits detectable at 24 h (Fig. 3i). In non-responders, no major decreases in procaspase-8 were detectable and only residual amounts of caspase-3 p21 were observed, which in U343 cells peaked transiently at ~4–8 h (Fig. 3j). The p21 subunit is the first cleavage product of procaspase-3 when processed by initiator caspases. In cells capable of entering the post-mitochondrial apoptosis execution phase, the p21 subunit is converted by caspase-3 autoprocessing into the p19 and p17 fragments, the fully mature, large caspase-3 subunits^27^.

Collectively, these results therefore indicate that while GBM cell lines show heterogeneous patterns of basal IAP expression, these do not differentiate between responders and non-responders. Interestingly, treatment-induced reductions in cIAPs in non-responders were associated residual, sub-lethal caspase activities after TRAIL/TL32711 treatment.

### The procaspase-8, procaspase-3, Bid signaling hub separates responder from non-responder cell lines

**Fig. 4:**
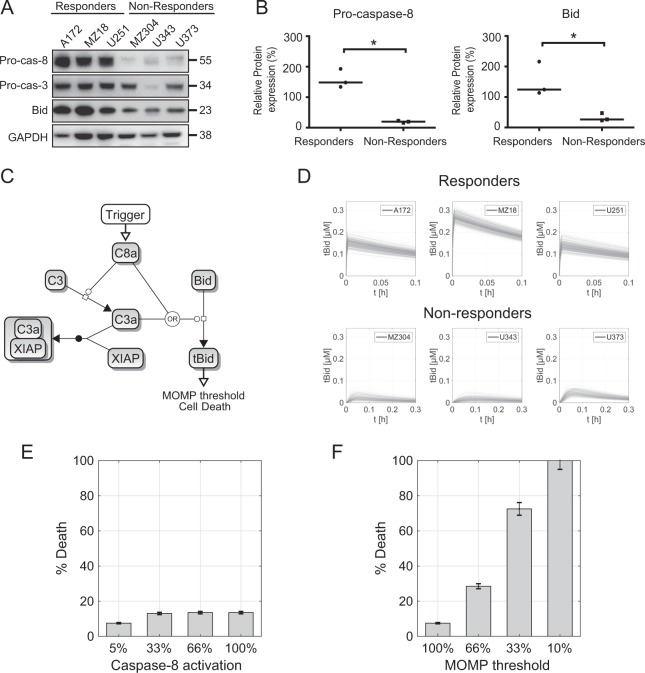
Mathematical modelling predicts lowering the MOMP threshold as a superior strategy to sensitize non-responder cell lines to TRAIL/TL32711. **a** Basal expression level of procaspase-8 and Bid proteins analysed from whole-cell lysates by western blotting. Actin served as loading control. **b** Protein amounts were quantified from original 12 bit grey scale images and compared between responder and non-responder cell lines. Lines represent medians. (* significant, Mann–Whitney *U*-test) **c** Topology of the mathematical model shown in systems biology graphical notation. Degradation processes were implemented but omitted here for clarity. Active caspase-8 served to trigger signaling. **d** Calculated tBid profiles for conditions of responder and non-responder cell lines. Taking estimates for cell-to-cell differences in protein expression amounts in account, *n* = 200 simulations were performed per cell line. **e** Predictions on the impact of enhanced caspase-8 activation on cell death in MZ304 cells. **f** Predictions on the impact of lowering the MOMP threshold in MZ304 cells. Bar graphs in **e**, **f** show means ± s.d., assuming 10% cell-to-cell heterogeneity in the efficacy of the sensitization strategy

To understand which molecular mechanisms might cause differential TRAIL/TL32711 responsiveness, we analysed the expression amounts of key signal transduction regulators of the extrinsic and intrinsic apoptosis pathways (TRAIL receptors 1 and 2, FADD, procaspase-8, cFLIP, Bid, Bcl-2, Bcl-xL, Mcl-1, Bax, Bak, cytochrome-c, Smac, Apaf-1, procaspase-9, XIAP, and procaspase-3) that have already been reported for these GBM cell lines^9^. From these comparisons and our own independent validation, we found that only procaspase-8 and Bid expression differed significantly, with both proteins expressed at substantially lower amounts in non-responders (Fig. 4a, b). The low expression of these two proteins, together with the evidence of residually activated caspase-3 in non-responders (Fig. 3j), indicates that the pro-apoptotic triad in which caspase-8 activates caspase-3, and in which both cleave Bid to truncated Bid (tBid)^28,29^, might not mount sufficient pro-apoptotic signals to trigger Bax/Bak-dependent mitochondrial outer membrane permeabilisation (MOMP) and apoptosis execution^30^.

To more formally test and validate this conclusion, we implemented a mathematical model of the caspase-8/caspase-3/Bid signalling triad, in which we also included the caspase-3 inhibitor XIAP, as a major limiter of MOMP-independent type I signal transduction (Fig. 4c). All reactions were assumed to obey the law of mass action and were parameterised with catalytic rate, binding and dissociation constants, the basal protein expression amounts found in the respective cell lines as well as the half-times of short lived reactants (see Methods and Supplementary Information 1 for details). We used this model to compare calculated profiles of tBid between responder and non-responder cell lines, expecting impaired tBid outputs in non-responder cell lines. Indeed, tBid amounts tBid remained significantly lower for non-responder conditions when triggering the model with active caspase-8 (Fig. 4d). From the differences between tBid profiles for responders and non-responders, we estimated the tBid ranges required to induce cell death, and predicted the efficacy of sensitization strategies. Within the context of the signalling hub studied here, these strategies included the enhancement of caspase-8 activation inputs or lowering the MOMP threshold in non-responder cell lines. As shown for the example of MZ304 cells, enhancing caspase-8 activation only modestly increased cell death responses (Fig. 4e), whereas decreasing the tBid amount required to trigger MOMP predicted a potentiation of cell death (Fig. 4f). Similar results were obtained for the other non-responder cell lines (not shown).

Taken together, these results indicate that enhancing signal inputs into the Caspase-8/Caspase-3/Bid triad would be expected to modestly increase cell death; whereas MOMP sensitization would be expected to potentiate cell death in otherwise TRAIL/TL32711-resistant GBM cell lines.

### Increasing mitochondrial apoptosis sensitivity by ABT-199 establishes TRAIL/TL32711 response synergies in otherwise TRAIL-resistant GBM cell lines

**Fig. 5:**
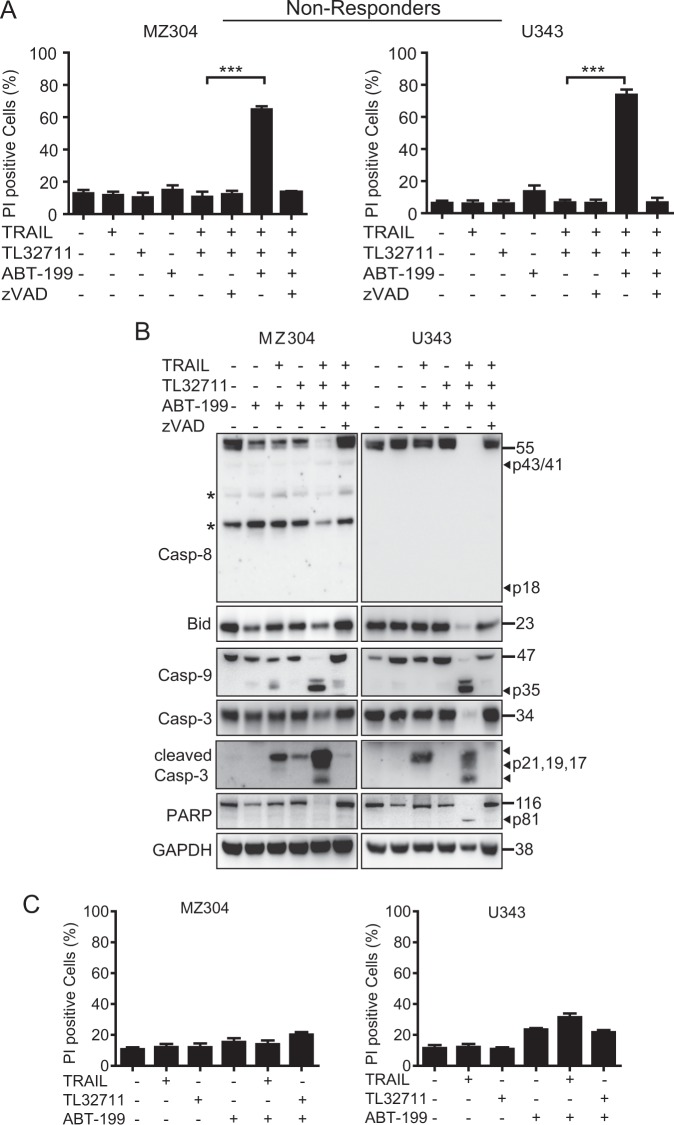
Increasing mitochondrial apoptosis sensitivity establishes TRAIL/TL32711 response synergies in otherwise TRAIL resistant GBM cell lines. **a** Cells were treated with TRAIL (100 ng/ml) and/or TL32711 (1 µM) in the presence or absence of ABT-199 (10 μM) and zVAD-fmk (50 μM). Cell death was measured after 24 h by PI-based flow cytometry. Data represent mean ± SEM of three independent experiments. ***p < 0.001 (one-way ANOVA followed by Tukey post hoc test). **b** Caspase processing and substrate cleavage in non-responder cell lines treated alone or in combination with ABT-199 (5 μM), TRAIL (100 ng/ml) and TL32711 (100 nM). zVAD-fmk was used at 50 μM. Whole-cell lysates were analysed for the indicated proteins by western blotting. GAPDH served as loading control. Asterisks indicate unspecific bands. Representative results from three independent experiments are shown. **c** Non-responders are resistant to combinations of TRAIL or TL32711 with ABT-199 (100 ng/ml; 1 µM; 5 μM). Cell death was measured after 24 h by PI-based flow cytometry. Data represent mean ± SEM of three independent experiments

Enhanced caspase-8 activation indeed poorly sensitized non-responder cell lines to the combination of TRAIL and IAP antagonist (Supplementary Fig. 2, Supplementary Information 2). Next, we therefore tested the mathematical prediction that increasing mitochondrial apoptosis sensitivity poses a superior strategy to evoke synergistic responses to TRAIL/TL32711 treatment. Anti-apoptotic Bcl-2 family members now are druggable targets, with the specific Bcl-2 antagonist venetoclax/ABT-199 being approved for the treatment of 17p deleted chronic lymphocytic leukaemia^31^. Indeed, we found that MZ304 and U343 cells, exposed to ABT-199, responded highly synergistically to TRAIL/TL32711, with up to 80% cell death observed (Fig. 5a). Cell death remained entirely apoptotic, since caspase inhibitor zVAD-fmk completely prevented PI positivity (Fig. 5a). Immunoblotting confirmed these results, since co-treatment with TRAIL/TL32711 significantly enhanced caspase-8, -9 and -3 processing as well as Bid and PARP cleavage in non-responders (Fig. 5b). Recovery of full length proteins upon addition of zVAD-fmk further confirmed caspase dependency of signal transduction (Fig. 5b). Additional experiments confirmed that response synergies relied on the combination treatment of TRAIL/TL32711, as single-agent combinations with ABT-199 failed to notably sensitize non-responders (Fig. 5c).

**Fig. 6:**
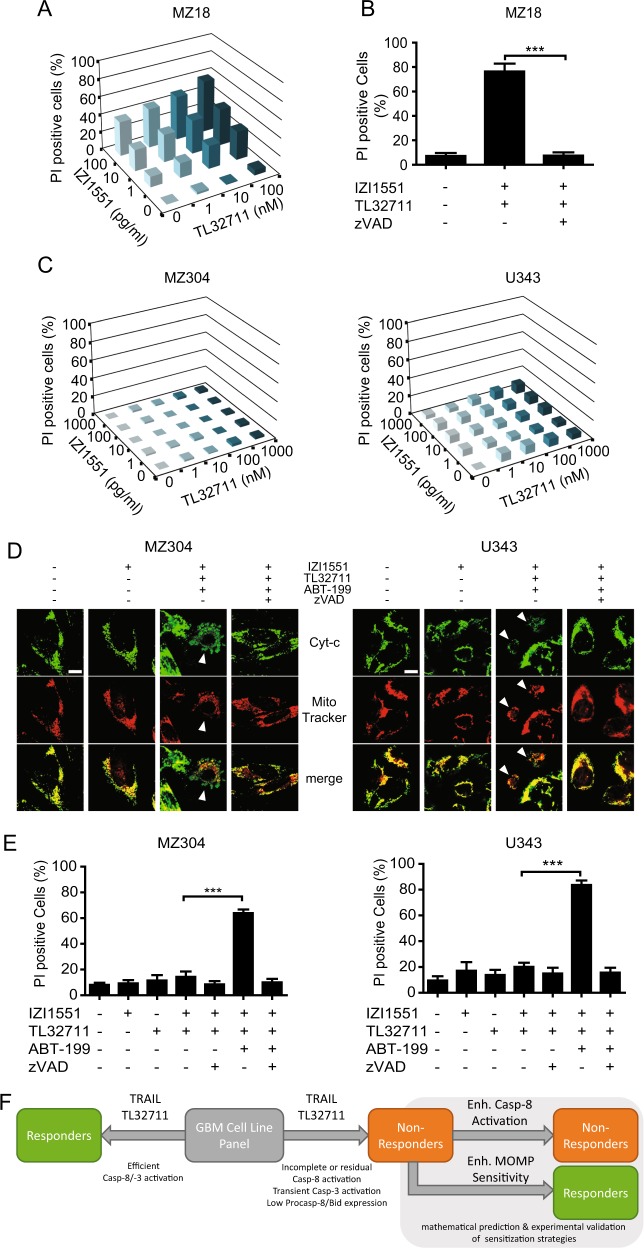
ABT-199 can sensitize non-responders to the combination of a translationally relevant, hexavalent TRAIL receptor agonist IZI1551 and TL32711. **a** Responder cell line MZ18 was treated with combinations of IZI1551 and TL32711. After 24 h, cell death was measured by PI-based flow cytometry. Cell death is shown as increase above untreated controls. Data are means of three independent experiments, with triplicate samples analysed in each experiment. Error bars were omitted for clarity. **b** MZ18 cells were treated with the combination of IZI1551/TL32711 in the presence or absence of pan-caspase inhibitor zVAD-fmk (50 µM) for 24 h. Cell death was measured by PI-based flow cytometry. Data represent mean ± SEM of three independent experiments. ***p < 0.001 (one-way ANOVA followed by Tukey post hoc test). **c** Non-responder cell lines remained largely resistant to the combined treatment of IZI1551 and TL32711. **d** Immunofluorescence-based detection of cytochrome *c* release. MZ304 cells were treated for 16 h, U343 cells were treated for 24 h (10 ng/ml IZI1551; 1 μM TL32711; 10 μM ABT-199; 50 μM zVAD-fmk). Scale bar 10 µm. Arrowheads indicate cells that released cytochrome *c*. **e** Cells were treated with IZI1551 (10 ng/ml) and/or TL32711 (1 µM) in the presence or absence of ABT-199 (5 μM) and zVAD-fmk (50 μM). Cell death was measured after 24 h by PI-based flow cytometry. Data represent mean ± SEM of three independent experiments. ***p < 0.001 (one-way ANOVA followed by Tukey post hoc test). **f** Graphical summary

Due to the shortcomings and limited clinical efficacy of previous TRAIL-based therapeutics, superior 2nd generation multivalent TRAIL-R ligands have been developed and have now entered phase I clinical studies, including trial segments making use of ABT-199 co-treatments (NCT03082209). We therefore tested whether our sensitization strategy can be transferred to treatments based on IZI1551, a hexavalent Fc-single-chain TRAIL (Fc-scTRAIL) format that has recently been extensively characterized^12^. As would be expected, IZI1551-induced potent response synergies with TL32711 in responder cell line MZ18 (Fig. 6a) and cell death was entirely apoptotic (Fig. 6b). Non-responders instead remained resistant to the combination of IZI1551/TL32711 (Fig. 6c), indicating that improved TRAIL receptor oligomerization by a multivalent ligand is insufficient to kill cells resistant to conventional recombinant TRAIL plus TL32711. However, response synergies could be provoked by co-treatment with ABT-199. IZI1551/TL32711/ABT-199 combination treatment-induced cytochrome-c release, whereas other single and combination treatments did not (Fig. 6d, and data not shown), and non-responders underwent apoptotic cell death (Fig. 6e). In contrast, human LUHMES brain stem cells were fully resistant to IZI1551/TL32711 and could not be sensitized by addition of high concentrations of ABT-199 (Supplementary Fig. 3).

These findings confirm the prediction that sensitizing non-responders downstream of the caspase-8/caspase-3/Bid triad is the most effective means to enhance apoptosis responsiveness in otherwise TRAIL/TL32711 resistant GBM cells. This provides a basis for case-specifically selecting between dual or triple treatments in order to enhance TRAIL-induced cell death responses.

## Discussion

In this study, we demonstrate that GBM cell lines respond heterogeneously to the combination of TRAIL and IAP antagonist TL32711. Apoptosis, the only observed cell death modality, was efficiently induced only in a subset of cell lines. In contrast to previous studies that described highly effective cell death induction by the combination of TRAIL receptor stimulation and IAP antagonism^8,32^, our results indicate that this treatment combination might frequently remain largely ineffective in GBM. Mathematical predictions followed by experimental studies demonstrated that otherwise TRAIL-resistant GBM cells respond synergistically towards combinations of TRAIL/TL32711 in presence of Bcl-2 antagonist ABT-199, indicating the need for triple treatment combinations (Fig. 6f).

TL32711, the bivalent IAP antagonist used in this study, is comparable to or even more potent than other IAP antagonists that have been used in pre-clinical studies or that have entered clinical trials, such as BV6, SM83 or LCL-161^33–35^. The discrepancies between our study and prior publications on the efficacy of TRAIL/IAP antagonist combinations therefore are unlikely to be attributable to the choice of IAP antagonist but rather might reflect that previous studies primarily focused on elucidating the molecular mechanisms of signal transduction in scenarios where response synergies manifest. As such, our results complement rather than contradict previous work.

Prior studies on the efficacy of combining TRAIL-R agonists and IAP antagonists in GBM made use of human recombinant TRAIL or TRAIL-R2-directed agonistic antibodies^8,32^. The cell death-inducing activity of recombinant TRAIL largely might be attributable to protein aggregates^10,36,37^. The potency of agonistic antibodies is limited by failing to sufficiently cluster TRAIL receptors, and in vivo efficacy appears to require Fcγ-receptor dependent aggregation in presence of immune cells^38^. Together with their short in vivo half times, hrTRAIL or monovalent agonistic antibodies provide little to no patient benefit^5,39^. In our study, we therefore also conducted experiments in which we used a translationally relevant 2nd generation TRAIL variant (IZI1551). This agonist of both TRAIL-R1 and -R2 is significantly more potent than prior ligands and was shown to possess superior in vivo pharmacokinetic and pharmacodynamic properties^12^. Similar multivalent TRAIL-R agonists now have entered phase I trials (NCT03082209). Interestingly, despite the superior potency of hexavalent agonists, cell lines resistant to the combination of TRAIL/TL32711 likewise failed to respond to IZI1551/TL32711. As the responder and non-responder cell lines used in this study do not differ significantly in their TRAIL-R expression^9^, the reason for cellular TRAIL/TL32711 resistance likely lies downstream of receptor activation and oligomerisation.

Overall, we can summarize that the response heterogeneity observed in our panel of cell lines is unlikely attributable to the choice of IAP antagonist or TRAIL receptor agonist but rather highlights the heterogeneity of the disease. For future translational studies, this flags the need to identify reliable predictors for treatment responsiveness and resistance, as well as effective interventions to enhance treatment responsiveness in cases where the combination of TRAIL/IAP antagonist is insufficient to induce cell death. It is noteworthy that the basal amounts of direct pharmacological targets, namely IAP proteins, are not indicative of TRAIL/TL32711 responsiveness. Similarly, IAP protein amounts do not predict responsiveness to single agent treatment with TL32711 or the combination with standard-of-care chemotherapeutic temozolomide in GBM^24^. While for TRAIL responsiveness, TRAIL-Rs obviously need to be present, also here the expression amounts apparently do not correlate with TRAIL responsiveness^9,40,41^.

We observed that non-responders failed to efficiently activate and process procaspase-8 in response to TRAIL/TL32711, indicating that upstream signal transduction might be impaired. In addition, non-responding cell lines expressed low amounts of procaspase-8 and Bid, associated with residual, non-lethal caspase-3 activation, indicating that downstream signalling towards apoptosis execution is likely impaired in these cells as well. Based on our current understanding of TRAIL DISC formation, IAP-antagonist-induced ripoptosome formation and subsequent signal transduction, it can be argued that the combination of TRAIL and IAP-antagonist is the most apical targeted pharmacological intervention currently possible, maximizing the cellular potential to form primary signalling hubs on which cell death-inducing signals can be mounted. With substantial evidence for on-target activity, the possible mechanisms for resistance must lie further downstream, though upstream of MOMP, as this is typically a point-of-no-return towards cell death execution. We found that ineffective signalling through the caspase-8/caspase-3/Bid hub is associated with TRAIL/TL32711 resistance. Predicted mathematically and subsequently confirmed experimentally, sensitizing mitochondria to MOMP restores response synergies. In contrast, reducing the amounts of cFLIP or RIP1, both of which can limit caspase-8 activation, only modestly sensitized non-responder cells. cFLIP isoforms, as catalytically inactivate caspase-8 homologs, might reduce caspase-8 activation or restrict caspase-8 activity to the DISC^42^. RIP1 complexes can form in response to IAP antagonist treatment and RIP1 is a natural component of TRAIL receptor complex 2, and can thereby relay signals towards NFκB signalling cascades as well as necroptosis. In select scenarios, RIP1 can also promote caspase-8 activation^43^. However, in our scenarios, RIP1 clearly played a protective role in non-responder cell lines, as its depletion enhanced caspase-dependent cell death. Consistent with this, in non-responders RIP1 and caspase-8 did not interact in the presence of IAP antagonists. As predicted by mathematical modelling, when signalling through the caspase-8/caspase-3/Bid hub is impaired, sensitizing mitochondria to MOMP allows otherwise sub-lethal caspase activation to effectively trigger the apoptosis execution phase in non-responders. Response synergies therefore require the triple treatment of TRAIL/TL32711/ABT199.

Future translational studies, testing combinations of TRAIL-based therapeutics and IAP antagonists, in for example, patient explant cultures, could elucidate if pre-treatment protein amounts of the caspase-8/caspase-3/Bid triad indicate treatment responsiveness. Additionally, such studies could investigate whether case-specific predictions could be made, possibly with the assistance of mathematical modelling, on whether the further addition of Bcl-2-family member antagonists to the treatment regimen could efficiently sensitize TRAIL/TL32711-resistant GBM tissues to treatment.

## Materials and methods

### Reagents and drugs

Recombinant human TRAIL was purchased from Peprotech, USA. ABT-199, zVAD-fmk and TL32711 were from Active Biochem, Hong Kong, China. Necrostatin was from Calbiochem/Merck, Germany. IZI1551 was produced at the Institute of Cell Biology and Immunology (University of Stuttgart, Germany), as described previously^12^. RIP1 and cFlip siRNAs were purchased from Sigma-Aldrich Ireland Ltd (Dublin, Ireland).

### Cell lines and culturing

GBM cell lines A172, U251, U343, and U373 are commercially available from ATCC. Low passage GBM cell lines MZ18 and MZ304 were kindly provided by Professor Donat Kögel of Johann Wolfgang Goethe University Hospital, Frankfurt, Germany. Cell lines were authenticated using DNA fingerprinting short-tandem repeat profiling and regularly tested for mycoplasma infections. GBM cells were cultured in Dulbecco’s modified Eagle’s medium (DMEM) supplemented with 10% heat-inactivated fetal calf serum, 100 U/ml penicillin and 100 mg/ml streptomycin and were maintained in a humidified incubator at 37 °C with 5% CO_2_. LUHMES cells were from ATCC and cultured in DMEM with 1% N_2_ supplement (Gibco) and 40 ng/ml b-FGF. For colony formation assays, 500 cells were plated, and colonies were determined after 10 days of growth.

### siRNA transfections

The siRNAs targeting RIP1 (target sequence #1: GACAUUUCCUGGCAUUGAA; target sequence #2: CAGCAAAGACCUUACGAGA), cFLIP (target sequence #1: GGGAUGUUGCAUAGAUGU; target sequence #2: CUCACCUUGUUUCGGACUA) and scrambled control siRNA (target sequence of AAUUCGGAAGAGCAGCUCC[dT]) were purchased from Sigma-Aldrich Ireland Ltd. Transfections were performed using lipofectamine®2000 (Invitrogen, Paisley, UK) according to the manufacturer’s protocol. Briefly, cells were transfected with 1 μg of siRNA for 4 h using lipofectamine®2000 in antibiotic-free Opti-MEM medium (Life Technologies, Paisley, Scotland). Subsequently, cells were kept in fresh DMEM for at least 24 h.

### Western blots analysis and reagents

To obtain whole-cell lysates, cells were washed with Hank’s buffered saline solution and lysed on ice with a lysis buffer containing 0.5 mM Tris-HCl (pH 6.8), 2% SDS (w/v), and protease inhibitor cocktails (Sigma-Aldrich Ireland Ltd). Protein concentrations were determined using a BCA protein assay kit (Pierce, Rockford, IL, USA). An equal amount of proteins (20 μg) were diluted with Laemmli loading buffer and were separated on 10–15% SDS-polyacrylamide gels. Proteins were then transferred to nitrocellulose membranes in transfer buffer (25 mM Tris, 192 mM glycine, 20% methanol (v/v), and 0.01% SDS) at 18 V for 90 min. Following transfer of the proteins, the nitrocellulose membranes were blocked with 5% non-fat dry milk in TBST (15 mM Tris-HCl, pH 7.5, 200 mM NaCl, and 0.1% Tween 20) at room temperature for 1 h. Following blocking, the membranes were incubated with antibodies: A mouse monoclonal caspase-8 antibody (ALX-804–242, Enzo Life Science, Ltd., Exeter, UK), a rabbit polyclonal caspase-3 antibody (#9662, Cell Signalling Technology, Danvers, MA, USA), a rabbit polyclonal caspase-9 antibody (#9502, Cell Signalling Technology, Danvers, MA, USA), a rabbit polyclonal PARP antibody (#9542 Cell Signalling Technology, Danvers, MA, USA), a rabbit polyclonal Bid antibody (#2002 Cell Signalling Technology, Danvers, MA, USA), a rabbit polyclonal XIAP antibody (#2042 Cell Signalling Technology, Danvers, MA, USA), a rabbit polyclonal cIAP1 antibody (# 4952 Cell Signalling Technology, Danvers, MA, USA), a rabbit polyclonal cIAP2 antibody (#3130 Cell Signalling Technology, Danvers, MA, USA), a rabbit polyclonal cFlip antibody (#AXL-804-428), a mouse monoclonal RIP1 antibody (#4926 Cell Signalling Technology, Danvers, MA, USA), a mouse monoclonal α-tubulin antibody (#T9026, Sigma-Aldrich), a mouse monoclonal β-actin antibody (A5441, Sigma-Aldrich). Mouse or rabbit (AP124P, AP132P, Millipore) IgG horseradish peroxidase-conjugated secondary antibodies were used at a dilution of 1:20,000. The membranes were washed with TBST three times for 5 min and incubated with secondary antibodies for 1 h. Proteins were then detected using enhanced chemiluminescence detection reagent (Amersham Biosciences) at 12-bit dynamic range using a Fuji LAS 4000 CCD system (Fujifilm UK, Bedfordshire, UK).

### Immunoprecipitation

Cells were pre-treated for 1 h with zVAD-fmk (50 μM), followed by adding TRAIL (100 ng/ml) in combination with TL32711 (1 μM) for 3 h. Cells were then lysed in RIPA buffer (Tris 50 mM, NaCl 150 mM, SDS 0.1%, NaDeoxycholate 0.5%, NP40 1%) supplemented with complete protease and phosphatase inhibitors cocktail. The cell lysates were then precleared with protein G magnetic Dynabeads for 2 h at 4 °C. The precleared lysates were incubated overnight at 4 °C with 5 μg of caspase-8 or IgG (ALX-804-242, Enzo Life Science, Ltd., Exeter, UK) antibody. Following the incubation period, the samples were washed once with lysis buffer followed by two washes with PBS. Finally, the immunoprecipitates were denatured by adding Laemmli buffer and boiled for 10 min at 70 °C. Western blotting was then performed as described above.

### Immunofluorescence staining and confocal microscopy

Cells were grown on microscope cover glasses coated with 2.5 µg/ml of Collagene R Solution (Serva, Heidelberg, Germany) and stained with 300 nM MitoTracker Deep Red FM (Invitrogen), followed by treatment with IZI1551, alone or in combinations with TL32711 and ABT-199 After treatment, cells were washed with PBS and fixed using 4% paraformaldehyde for 30 min. Cells were permeabilised with 0.1% Triton X-100 in PBS and blocked with 4% Bovine Serum Albumin (BSA) before incubation with 5 μg/ml of monoclonal cytochrome *c* antibody (Clone 6H2.B4; Thermo Fisher Scientific) in 2% BSA/PBS. After incubation at room temperature for 1 h, cells were washed three times with PBS and incubated with 4 μg/ml of Alexa Fluor 488-labeled secondary antibody (#A-11029, Thermo Fisher Scientific) for 45 min. After 3 PBS washes, cells were mounted (Fluoromount-G, SouthernBiotech). Images were captured using a confocal laser scanning microscope (LSM 710, Carl Zeiss) equipped with a Plan Apochromat ×63/1.40 DIC M27 (Carl Zeiss, Jena, Germany) oil-immersion objective. MitoTracker was excited with a 633 nm laser and its emission was detected from 638 to 759 nm. Alexa Fluor 488 was excited with a 488 nm laser and detected with a 496/602 emission filter. Secondary antibody controls did not provide signal above background noise. Image processing and analysis was performed with Zen black 2.1 software (Carl Zeiss).

### Cell death analysis by flow cytometry

Cell death was measured using a BD LSRII flow cytometer (Oxford, UK). Cells were seeded at a density of 5000 cells per well in 96-well plates and were allowed to adhere overnight. TRAIL, TL32711 and combination treatments were performed for 24 h. Cell death was assessed by uptake of propidium iodide (PI) (1 µg/ml, 10 min incubation at room temperature in the dark). PI was excited at 561 nm and fluorescence emission was collected through a 605/40 nm band-pass filter. Data was acquired in.fcs file format and analysed using Cyflogic software (Version 1.2.1, CyFlo, Turku, Finland).

### TNF-α ELISA

A human TNF-α ELISA Kit (Enzo Life Sciences, UK) was used to determine whether cells secrete TNF-α following treatment. Cells were treated with TRAIL (100 ng/mL), TL32711 (1 µM) or in combination for 24 h. Samples were processed and analysed according to the manufacturer’s instructions.

### Statistical analysis and mathematical modelling

Statistical analyses were performed using GraphPad prism software version 5 (GraphPad software Inc., La Jolla, CA, USA). Statistical tests are detailed in the respective figure legends (*p*-values ≤0.05 were considered statistically significant). Responses to TRAIL and TL32711 were analysed for synergy by Webb’s fractional product method^44^. Mathematical modelling was performed with the IQM toolbox [http://www.intiquan.com/iqm-tools] in MATLAB (R2017a). Detailed information on the model implementation and parameterisation are provided as supplementary information 2.

## Electronic supplementary material

Supplemental Material Model

Supplemental Material Fig1

Supplemental Material results text

Supplemental Material Fig2

Supplemental Material Fig3

Supplementary figure legends
