# Supplementary material for: Sensitization of glioblastoma cells to TRAIL-induced apoptosis by IAP- and Bcl-2 antagonism
Source: Cell Death Dis. 2018 Nov 1;9(11):1112. doi: 10.1038/s41419-018-1160-2 (PMC6212537; doi:10.1038/s41419-018-1160-2)
Supplement: Supplementary file 1 — Supplemental Material Model [file 41419_2018_1160_MOESM1_ESM.doc]

# Supplementary Information 2

for

**The triple treatment combination of TRAIL/TL32711/ABT-199 is required to eliminate highly resistant glioblastoma cells by apoptosis**

By Frank A Lincoln, Dirke Imig, Chiara Boccellato, Viktorija Juric, Janis Noonan, Roland E Kontermann, Frank Allgöwer, Brona M Murphy, Markus Rehm

**The modelled network**

The mathematical model (Fig.4C) represents the core reactions of the crucial interplay between caspase-8, caspase-3 and Bid, where activated caspase-8 can activate caspase-3 and both caspases can activate Bid. XIAP was implemented as the primary cellular inhibitor of caspase-3. Since the model here was used in the context of co-treatments with IAP antagonist TL32711, the latter was implemented to antagonise the function of XIAP. Activated caspase-8 serves to trigger the network, whereas the amounts of truncated Bid (Bid cleaved by caspases-8 or -3) serve as an output that was measured against the threshold required to induce mitochondrial outer membrane permeabilisation (MOMP) and apoptosis execution. Protein degradation was implemented for all processed/activated protein species and complexes forming as a consequence of triggering the network. All reactions were based on mass action kinetics. Detailed overviews for all reactions and their parameterisation is provided in Tables 1-4 in this document.

## Mathematical implementation

For model implementation and simulation the IQM toolbox [<http://www.intiquan.com/iqm-tools>] was used with MATLAB (The MathWorks, Inc., Natick), release 2017a. A set of ordinary differential equations describes the network structure. The modeled reactions are listed in Table 3. Within isogenic cell populations, protein expression amounts were assumed to be log-normally distributed, using mean expression amounts determined experimentally (Table 2) and assuming an s.d. of 20 nM. 200 independent samples were drawn for each cell line from the underlying protein distributions, representing a population of different cells. Profiles of tBid over time (Fig.2D) were simulated for the sampled initial protein amounts and with reaction parameter values as shown in Table 4. For responder cell lines, which experimentally were shown to efficiently activate caspase-8, inputs of least 40% activated capsase-8 were tested. For non-responder cell lines, which poorly activate caspase-8, inputs of 5% active caspase-8 were modeled. Amounts of active caspase-8 inputs were modified as part of simulations to test sensitization strategies. Thresholds required for efficient MOMP induction were determined from the range between tBid amounts reached between responder and non-responder cell lines. 10% cell-to-cell heterogeneity in MOMP thresholds were assumed based on prior studies of TRAIL-induced MOMP induction ^1^.

**Table 1:** Abbreviations of proteins and complexes in the mathematical model.

| **Nr.** |  | **Symbol** | **Explanation** |
| --- | --- | --- | --- |
| 1 |  | C8 | procaspase-8 |
| 2 |  | C8a | active caspase-8 |
| 3 |  | C3 | procaspase-3 |
| 4 |  | C3a | active caspase-3 |
| 5 |  | Bid | BH3 interacting-domain death agonist |
| 6 |  | tBid | truncated Bid |
| 7 |  | XIAP | X-linked inhibitor of apoptosis protein |
| 8 |  | XIAP_C3a | complex of active caspase-3 and XIAP |
| 9 |  | TL | TL32711 |
| 10 |  | XIAP_TL | complex of TL32711 and XIAP |

**Table 2:** Initial mean protein concentrations.

| **Protein** | Initial concentration [µM] | | | | | | Comment |  |
| --- | --- | --- | --- | --- | --- | --- | --- | --- |
|  | A172 | MZ18 | U251 | MZ304 | U343 | U373 |  |  |
| C8 | 0.491 | 0.377 | 0.341 | 0.05 | 0.057 | 0.041 | Own data, compared and normalized to previously published data of Hela cells in ^2^ | |
| C3 | 0.203 | 0.295 | 0.128 | 0.19 | 0.01 | 0.103 | Own data, compared and normalized to previously published data of Hela cells in ^3^ | |
| Bid | 0.155 | 0.27 | 0.142 | 0.033 | 0.029 | 0.059 | Own data, compared and normalized to previously published data in ^4^ | |
| XIAP | 0.024 | 0.061 | 0.04 | 0.113 | 0.057 | 0.054 | Data previously published in published data in ^4^ | |
| TL | 1000 | | | | | | Maximum treatment concentration | |

**Table 3:** Model reaction network.

| # |  | | Reaction velocity |  |
| --- | --- | --- | --- | --- |
|  |  |  |  | |
| 1 | C3 → | C3a | v(1) =k1*C8a*C3a | |
| 2 | Bid → | tBid | v(2) = k2*Bid*C8a | |
| 3 | Bid → | tBid | v(3) = k3*Bid*C3a | |
| 4 | C3a + XIAP → | XIAP_C3a | v(4) = k4*C3*XIAP | |
| 5 | XIAP_C3a → | C3a + XIAP | v(5) = k5*XIAP_C3a | |
| 6 | XIAP + TL → | XIAP_TL | v(6) = k6*XIAP*TL | |
| 7 | XIAP_TL → | XIAP + TL | v(7) = k7*XIAP_TL | |
| 8 | C8a → |  | v(8) = d1*C8a | |
| 9 | tBid → |  | v(9) = d2*tBid | |
| 10 | C3a → |  | v(10) = d3*C3a | |
| 12 | XIAP_C3a → |  | v(12) = d4*XIAP_C3a | |

Table 4: Kinetic constants and degradation rates in the model.

|  | **Parameter** | **k^a)^** | **k^b)^** | **References** |
| --- | --- | --- | --- | --- |
| k1 | activation of procaspase-3 by caspase-8 | 52.2 |  | 5 |
| k2 | truncation of Bid by active caspase-8 | 104.4 |  | ^6^ indicates that Bid is cleaved faster by C8a than C3 is cleaved by C8a. |
| k3 | truncation of Bid by active caspase-3 | 52.2 |  | ^6^ indicates that cleavage of Bid is better by C8a than by C3a. |
| k4 | complex formation of C3a and XIAP | 156 |  | 7 |
| k5 | binding of cFlip to the DISC |  | 0.144 | 7 |
| k6 | binding of TL to XIAP | 0.0045 |  | adapted to match, together with k7, the reported *K*_D_ (45 nM) ^8^ |
| k7 | dissociation of TL_XIAP complex |  | 0.1 | adapted to match, together with k6, the reported *K*_D_ (45 nM) ^8^ |
| d1 | degradation of C8a |  | 8.8e-3 | estimated from results shown in ^9^ |
| d2 | degradation of tBid |  | 6.9e-2 | half life assumed as approx. 10 min (educated guess) |
| d3 | degradation of C3a |  | 5.8e-3 | 10 |
| d4 | degradation of XIAP_C3a |  | 3.47e-2 | 11 |

Units: a) µM-1 min-1 b) min-1

**References**

1. Hellwig CT, Kohler BF, Lehtivarjo AK, Dussmann H, Courtney MJ, Prehn JH, et al. Real time analysis of tumor necrosis factor-related apoptosis-inducing ligand/cycloheximide-induced caspase activities during apoptosis initiation. J Biol Chem 2008, 283(31): 21676-21685.

2. Laussmann MA, Passante E, Hellwig CT, Tomiczek B, Flanagan L, Prehn JH, et al. Proteasome Inhibition Can Impair Caspase-8 Activation upon Submaximal Stimulation of Apoptotic Tumor Necrosis Factor-related Apoptosis Inducing Ligand (TRAIL) Signaling. J Biol Chem 2012, 287(18): 14402-14411.

3. Rehm M, Huber HJ, Dussmann H, Prehn JH. Systems analysis of effector caspase activation and its control by X-linked inhibitor of apoptosis protein. Embo J 2006, 25(18): 4338-4349.

4. Weyhenmeyer BC, Noonan J, Wurstle ML, Lincoln FA, Johnston G, Rehm M, et al. Predicting the cell death responsiveness and sensitization of glioma cells to TRAIL and temozolomide. Oncotarget 2016, 7(38): 61295-61311.

5. Stennicke HR, Jurgensmeier JM, Shin H, Deveraux Q, Wolf BB, Yang X, et al. Pro-caspase-3 is a major physiologic target of caspase-8. J Biol Chem 1998, 273(42): 27084-27090.

6. Fischer U, Stroh C, Schulze-Osthoff K. Unique and overlapping substrate specificities of caspase-8 and caspase-10. Oncogene 2006, 25(1): 152-159.

7. Riedl SJ, Renatus M, Schwarzenbacher R, Zhou Q, Sun C, Fesik SW, et al. Structural basis for the inhibition of caspase-3 by XIAP. Cell 2001, 104(5): 791-800.

8. Allensworth JL, Sauer SJ, Lyerly HK, Morse MA, Devi GR. Smac mimetic Birinapant induces apoptosis and enhances TRAIL potency in inflammatory breast cancer cells in an IAP-dependent and TNF-alpha-independent mechanism. Breast Cancer Res Treat 2013, 137(2): 359-371.

9. Gonzalvez F, Lawrence D, Yang B, Yee S, Pitti R, Marsters S, et al. TRAF2 sets a threshold for extrinsic apoptosis by tagging caspase-8 with a ubiquitin shutoff timer. Mol Cell 2012, 48(6): 888-899.

10. Eissing T, Conzelmann H, Gilles E D, Allgöwer F, Bullinger E, Scheurich P. Bistability analyses of a caspase activation model for receptor-induced apoptosis.. J Biol Chem 2004, 279(35): 36892-7.

11. Yoo S J, Huh J R, Muro I, Yu H, Wang L, Wang S L, Feldman R M, Clem R J, Muller H A, Hay B A. Hid, Rpr and Grim negatively regulate DIAP1 levels through distinct mechanisms. Nat Cell Biol 2002, 4(6): 416-424.
