# Supplementary figures and images for: Sensitization of glioblastoma cells to TRAIL-induced apoptosis by IAP- and Bcl-2 antagonism
Source: Cell Death Dis. 2018 Nov 1;9(11):1112. doi: 10.1038/s41419-018-1160-2 (PMC6212537; doi:10.1038/s41419-018-1160-2)

Supplemental Fig.1

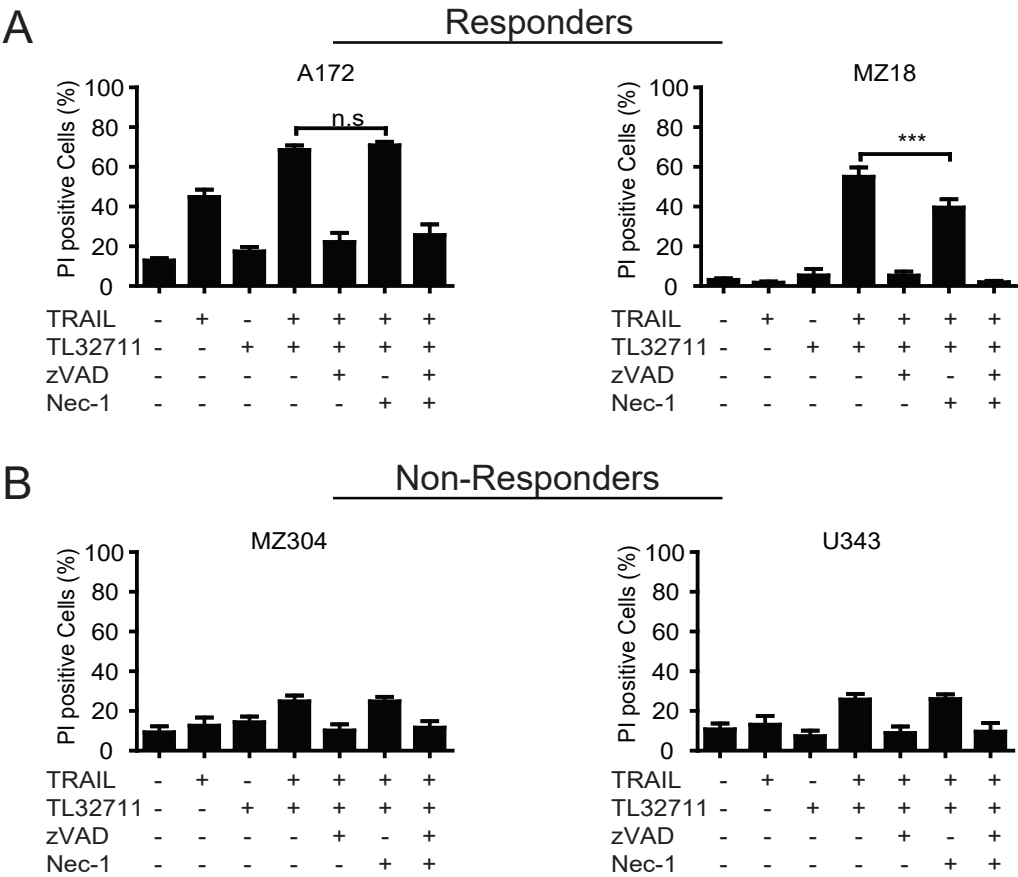

Supplement: Supplementary file 2 — Supplemental Material Fig1 [file 41419_2018_1160_MOESM2_ESM.pdf]

A

## Responders

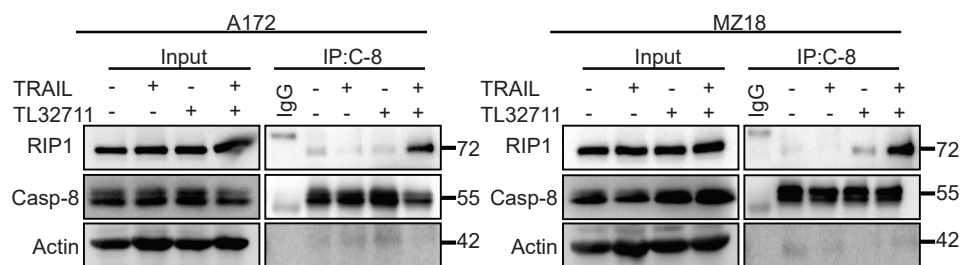

B

## Non-Responders

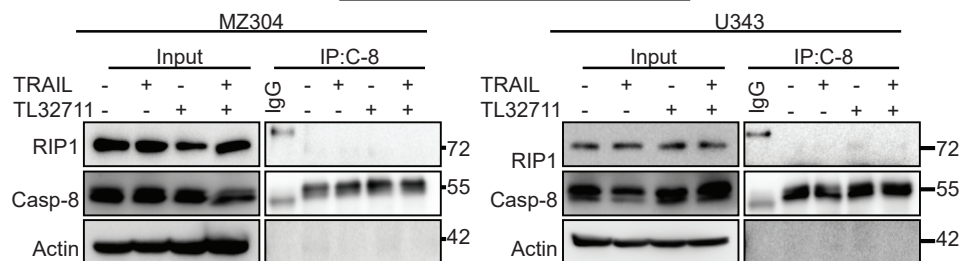

C

## Responders

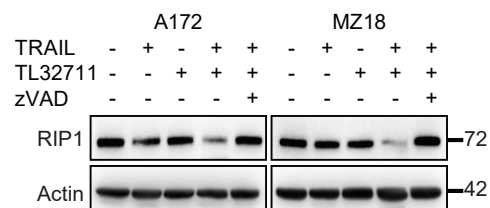

D

## Non-Responders

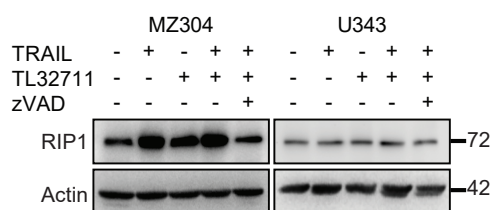

E

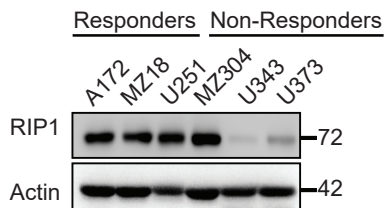

F

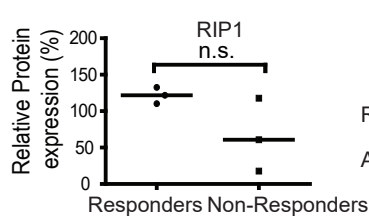

G

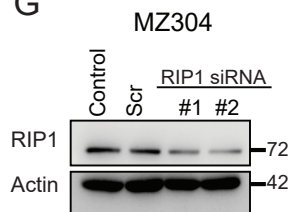

H

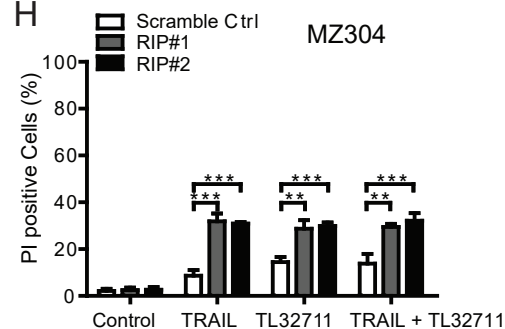

I

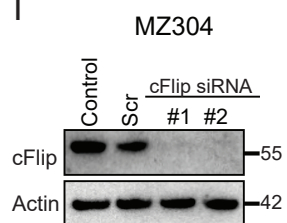

J

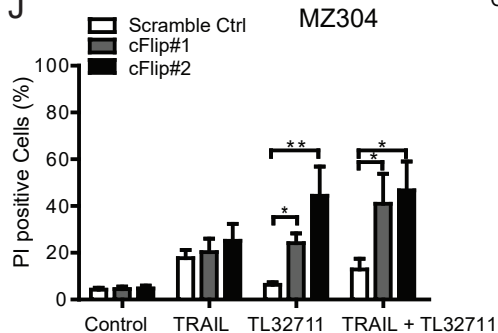

Supplement: Supplementary file 4 — Supplemental Material Fig2 [file 41419_2018_1160_MOESM4_ESM.pdf]

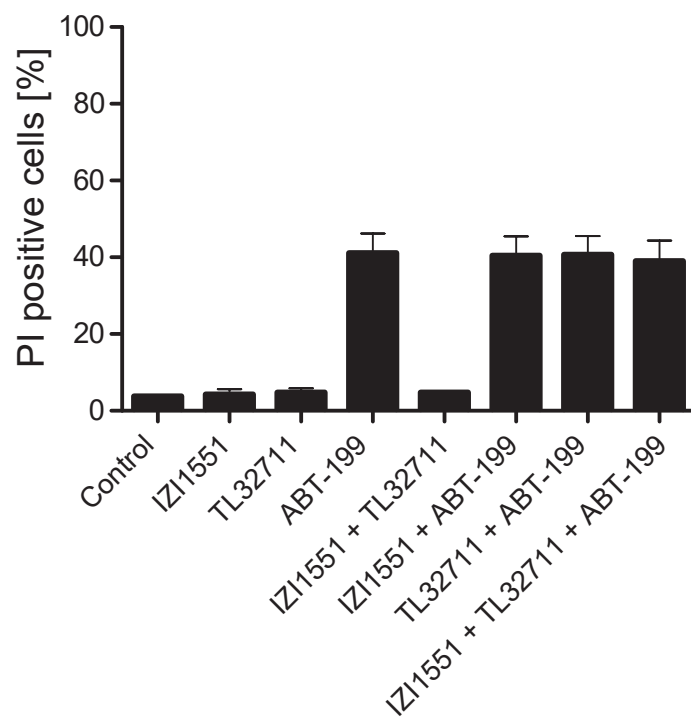

Supplement: Supplementary file 5 — Supplemental Material Fig3 [file 41419_2018_1160_MOESM5_ESM.pdf]
