# Supplementary material for: Sensitization of glioblastoma cells to TRAIL-induced apoptosis by IAP- and Bcl-2 antagonism
Source: Cell Death Dis. 2018 Nov 1;9(11):1112. doi: 10.1038/s41419-018-1160-2 (PMC6212537; doi:10.1038/s41419-018-1160-2)
Supplement: Supplementary file 3 — Supplemental Material results text [file 41419_2018_1160_MOESM3_ESM.docx]

**Supplemental Results: Depleting RIP1 or cFLIP promotes apoptosis in non-responders but is insufficient to cause response synergies to TRAIL/TL32711 treatment.**

We experimentally tested if promoting caspase-8 activation indeed would be limited in its efficacy to sensitize non-responder cell lines to the combination of TRAIL and IAP antagonist. To this end, targeted possible inhibitors of caspase-8 activation, such as RIP1 and cFLIP. RIP1 can relay signals that otherwise would result in the activation of caspase-8 towards the necroptosis signaling cascade as well as towards signaling pathways that promote the production of cytokines [^31^](#_ENREF_31)^,^ [^32^](#_ENREF_32). Interestingly, RIP1, as a scaffold protein of the ripoptosome or complex IIa, can also contribute to caspase-8 dependent death, especially when cIAPs are eliminated [^33^](#_ENREF_33). Since TL32711-induced cIAP inhibition is inefficient in non-responders, we hypothesized that non-responders possibly are incapable of forming ripoptosomes and that RIP1 functions primarily in a cell-protective manner in a TRAIL/TL32711 treatment setting. Indeed, in contrast to responder cell lines, immunoprecipitation of caspase-8 failed to co-immunoprecipitate RIP1 in non-responders in single or combination treatments with TRAIL and TL32711 (Supplemental Fig.2A,B). In responder cell lines, the amounts of RIP1 dropped upon treatment with TRAIL/TL32711 (Supplemental Fig.2C). This loss was prevented in presence of caspase inhibitor zVAD-fmk (Supplemental Fig.2C), indicating that caspase activation is sufficiently high to induce substantial RIP1 cleavage. In contrast, non-responders failed to cleave RIP1 or, as is the case for MZ304 cells, even increased RIP1 expression (Supplemental Fig.2D). We therefore studied if RIP1 expression or activity contributes to preventing efficient apoptosis induction in these cells. RIP1 was found to be expressed heterogeneously in non-responders, with only MZ304 cells expressing RIP1 amounts similar to those of responder cell lines (Supplemental Fig.2E,F). siRNA-based RIP1 depletion in MZ304 cells enhanced TRAIL and TL32711 sensitivity, but did not result in synergistic responses to the TRAIL/TL32711 combination (Supplemental Fig.2G,H). These sensitization results are therefore consistent with the predictions made by mathematically modeling. As shown in Supplemental Figure 1, inhibiting RIP1 activity by necrostatin did not notably affect cell death induction. To further validate the prediction that enhanced signaling into the triad would have limited potential in sensitizing non-responders, we also depleted cFLIP, an inactive capase-8 homolog that can interfere with caspase-8 dimerisation and activation [^11^](#_ENREF_11). While depletion of cFLIP sensitized MZ304 cells to TL32711 single-agent treatment, synergies in co-treatment with TRAIL were not detectable (Supplemental Fig.2I,J). Taken together, these findings therefore confirm that, as predicted by mathematical modeling, enhancing signaling into the triad of caspase-8, -3 and Bid has limited potential in enhancing the responsiveness of non-responders.
