# Supplementary material for: Sensitization of glioblastoma cells to TRAIL-induced apoptosis by IAP- and Bcl-2 antagonism
Source: Cell Death Dis. 2018 Nov 1;9(11):1112. doi: 10.1038/s41419-018-1160-2 (PMC6212537; doi:10.1038/s41419-018-1160-2)
Supplement: Supplementary file 6 — Supplementary figure legends [file 41419_2018_1160_MOESM6_ESM.docx]

**Supplemental Figure 1: RIP1 activity is largely dispensable for cell death induced by TRAIL/TL32711 in GBM cells.**

**(A,B)** Responders (A) or non-responders (B) were pre-treated with necrostatin 1 (Nec-1) (5 μM) and/or zVAD-fmk for 1 h prior to adding TRAIL (100 ng/ml) and/or TL32711 (1 µM). Cell death was measured by PI-based flow cytometry after 24 h of treatment. Data represent mean ± SEM of three independent experiments. ***p < 0.001 (one-way ANOVA followed by Tukey post-hoc test).

**Supplemental Figure 2: Depleting RIP1 or cFLIP promotes apoptosis in non-responders but is insufficient to cause synergistic responses to TRAIL/TL32711 treatment.**

**(A,B)** Caspase-8:RIP1 interaction in responder and non-responder cell lines. Cells were pre-treated with zVAD-fmk (50 µM) for 1 h after which TRAIL and/or TL32711 were added for 3 h (responders: 100 ng/ml TRAIL, 100 nM TL32711; non-responders: 100 ng/ml TRAIL, 1 µM TL32711). Caspase-8:RIP1 association was tested by immunoprecipitation. **(C,D)** Treatment-induced changes in RIP1 amounts in responders and non-responders. RIP1 amounts were determined from whole cell lysates by western blotting. Actin served as a loading control. **(E)** Basal expression level of RIP1 analyzed from whole cell lysates by western blotting. Actin served as loading control. **(F)** Protein amounts were quantified from original 12 bit gray scale images and compared between responder and non-responder cell lines. Lines represent medians. (n.s., not significant; Mann Whitney U-test). **(G)** MZ304 cells were transfected with scrambled siRNA (Scr) or RIP1 siRNAs. Depletion of RIP1 was assessed by western blotting. Actin served as loading control. **(H)** MZ304 cells transfected as indicated were exposed to TRAIL (100 ng/ml) and/or TL32711 (1 µM). Cell death was measured by PI-based flow cytometry 24 h after drug addition. **(I)** MZ304 cells were transfected with scrambled siRNA (Scr) or cFLIP siRNAs. Depletion of cFLIP was assessed by western blotting. Actin served as loading control. **(J)** MZ304 cells transfected as indicated were exposed to TRAIL (100 ng/ml) and/or TL32711 (1 µM). Cell death was measured by PI-based flow cytometry 24 h after drug addition. Data represent mean ± SEM of three independent experiments.

* p < 0.05; ** p < 0.01; *** p < 0.001 (two-way ANOVA followed by Tukey post-hoc test).

**Supplemental Figure 3: TRAIL resistance and absence of response synergies in human brain stem cells.**

LUHMES cells were treated as indicated for 24 h and cell death was measured by propidium iodide uptake. Cells were fully resistant to IZI1551/TL32711 and, despite notable intrinsic sensitivity to ABT-199, response synergies did not manifest. Data show means +/- s.d.
